# Detection of femoropopliteal arterial steno-occlusion at MR angiography: initial experience with artificial intelligence

**DOI:** 10.1186/s41747-024-00433-5

**Published:** 2024-03-13

**Authors:** Tri-Thien Nguyen, Lukas Folle, Thomas Bayer

**Affiliations:** 1 Institute of Neuroradiology and Radiology, Klinikum Fürth, Fürth, Germany; 2 Faculty of Pattern Recognition, Friedrich-Alexander-University Erlangen-Nuremberg, Erlangen, Germany; 3 Department of Radiology, University Hospital Erlangen, Friedrich-Alexander-University Erlangen-Nuremberg, Erlangen, Germany

**Keywords:** Artificial intelligence, Deep learning, Digital subtraction angiography, Magnetic resonance angiography, Peripheral arterial disease

## Abstract

**Background:**

This study evaluated a deep learning (DL) algorithm for detecting vessel steno-occlusions in patients with peripheral arterial disease (PAD). It utilised a private dataset, which was acquired and annotated by the authors through their institution and subsequently validated by two blinded readers.

**Methods:**

A single-centre retrospective study analysed 105 magnetic resonance angiography (MRA) images using an EfficientNet B0 DL model. Initially, inter-reader variability was assessed using the complete dataset. For a subset of these images (29 from the left side and 35 from the right side) where digital subtraction angiography (DSA) data was available as the ground truth, the model’s accuracy and the area under the curve at receiver operating characteristics analysis (ROC-AUC) were evaluated.

**Results:**

A total of 105 patient examinations (mean age, 75 years ±12 [mean ± standard deviation], 61 men) were evaluated. Radiologist-DL model agreement had a quadratic weighted Cohen κ ≥ 0.72 (left side) and ≥ 0.66 (right side). Radiologist inter-reader agreement was ≥ 0.90 (left side) and ≥ 0.87 (right side). The DL model achieved a 0.897 accuracy and a 0.913 ROC-AUC (left side) and 0.743 and 0.830 (right side). Radiologists achieved 0.931 and 0.862 accuracies, with 0.930 and 0.861 ROC-AUCs (left side), and 0.800 and 0.799 accuracies, with 0.771 ROC-AUCs (right side).

**Conclusion:**

The DL model provided valid results in identifying arterial steno-occlusion in the superficial femoral and popliteal arteries on MRA among PAD patients. However, it did not reach the inter-reader agreement of two radiologists.

**Relevance statement:**

The tested DL model is a promising tool for assisting in the detection of arterial steno-occlusion in patients with PAD, but further optimisation is necessary to provide radiologists with useful support in their daily routine diagnostics.

**Key points:**

• This study focused on the application of DL for arterial steno-occlusion detection in lower extremities on MRA.

• A previously developed DL model was tested for accuracy and inter-reader agreement.

• While the model showed promising results, it does not yet replace human expertise in detecting arterial steno-occlusion on MRA.

**Graphical Abstract:**

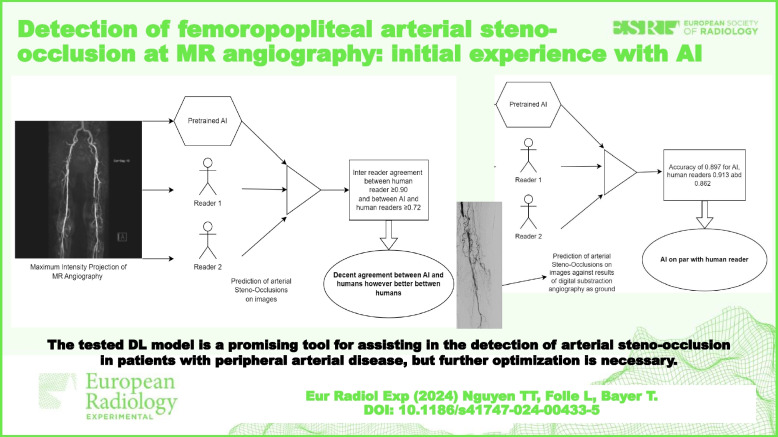

## Background

Peripheral arterial disease (PAD) of the lower extremities is a medical condition characterised by the narrowing or steno-occlusions of arteries that supply blood to the legs. Globally, the prevalence of this disease among individuals aged 25 years and above is approximately 5.6%, albeit with regional variations [1]. While femoropopliteal PAD mainly affects the arteries above the knee, lower leg PAD involves steno-occlusions below the knee. This disease poses a significant financial burden on the healthcare sector [2]. 

Magnetic resonance angiography (MRA) and computed tomography angiography (CTA) both offer comparable diagnostic capabilities in detecting PAD [3]. MRA provides robust results for visualising vessel steno-occlusions and informing clinical decisions, such as the choice between surgical bypass and interventional radiological approaches [4]. One significant advantage of MRA over CTA is that it does not involve the use of ionising radiation and does not require iodinated contrast material, which can be beneficial for certain patient groups. Despite this, digital subtraction angiography (DSA) remains the reference standard for imaging diagnosis in PAD, although its clinical value has been challenged due to potential complications associated with its invasive nature [5]. In addition to MRA and CTA, ultrasound also serves as another noninvasive imaging technique [4]. The common method for interpreting images from MRA is based on an analysis of maximum intensity projections (MIP) by one or more radiologists. However, this type of analysis, which includes the description of findings, can be both time-consuming and error-prone, depending on the quality of the diagnostic images.

Artificial intelligence (AI) is a broad field of computer science focused on creating machines that can perform tasks that typically require human intelligence. Deep learning, a subset of AI, uses neural networks, especially deep neural networks with many layers, to analyse various forms of data, recognise patterns, and make decisions [6]. These AI techniques have shown promising results across various medical and nonmedical domains and hold the potential to provide valuable support to radiologists. In the field of radiology, AI already plays a significant role in the detection of breast cancer [7] and in screening for lung tumours [8]. AI techniques can enhance productivity, reduce radiologists’ workload, and increase the objectivity of findings by mitigating inter-reader variability [9].

However, the application of AI methods to PAD in the lower extremities is still in its nascent stages. To our knowledge, only two studies have been published that use a deep learning (DL) approach for detecting arterial steno-occlusions in the lower limbs [10, 11]. Dai et al. [10] conducted a study on steno-occlusion detection using small, segmented areas of axial CTA slices. In a preliminary study by our own institution, a neural network was trained to detect arterial steno-occlusions in the thigh using MRA images and a private dataset, which was acquired by the authors through their institution [11], albeit without the foundation of a sufficiently large clinical dataset.

Therefore, the aim of this study was to evaluate, as an initial experience, whether the DL model published by Nguyen et al. [11] for detecting arterial steno-occlusions on MRA images would yield valid results in a clinical setting. This evaluation was conducted using a dual reader strategy.

## Methods

This retrospective, single-centre study was initiated upon receipt of ethical approval from the governing institution (Ethics Committee of Friedrich-Alexander-Universität Erlangen-Nürnberg, application number 21-366-Br).

### Dataset

Images for this study were sourced from 105 patient examinations conducted between 2017 and 2021 at Klinikum Fürth, Fürth, Germany. This timeframe was selected to ensure that the data had not been previously used in the model’s training process, as described by Nguyen et al. [11]. The study included both male and female participants aged 18 years and above, all symptomatic of PAD. However, patients with previous amputations were excluded. The study cohort comprised 61 men and 44 women, with ages ranging from 18 to 96 years. The mean age was approximately 75 years, with a standard deviation of 12 years.

**Fig. 1:**
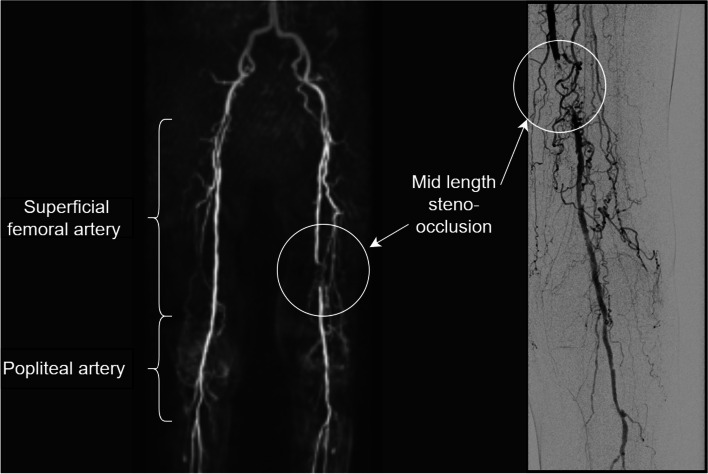
Exemplary view of the radial maximum intensity projection (left) with denoted pathology and according to digital subtraction angiography (right)

For this research, the focus was on three-dimensional radial MIP images of the upper legs, specifically those illustrating the superficial femoral and popliteal arteries, as shown in Fig. 1. The decision to limit the scope of the included image data aimed to achieve an optimal balance between essential image information and data size, thereby minimising hardware requirements. Consistent with the constraints of the pretrained model as detailed in Nguyen et al. [11], images of the lower legs were excluded from this study.

Only significant steno-occlusions, specifically those exceeding 50% in the visualised superficial femoral and popliteal arteries, were labelled. Radiologists T.N. and T.B., with 5 and 18 years of clinical experience, respectively, conducted a blinded assessment of the arteries’ steno-occlusion status. The evaluation utilised a 4-point scale (0-to-3), where labels ranged from 0 (indicating no steno-occlusion) to 3 (indicating long steno-occlusion). An additional label, 4, was used to denote unusable data, as detailed in Table 1. The thresholds for the different classes were determined by the authors based on clinical discretion, aiming to provide supplemental information on severity that impacts treatment decisions.

**Table 1:** Labelling scheme for assessing steno-occlusions

Score	Description
0	No steno-occlusion
1	Focal steno-occlusion < 0.5 cm
2	Mid-length/multiple focal steno-occlusions
3	Long steno-occlusion > 3 cm
4	Not usable (stents, bypass, heavy artifacts)

Samples that were deemed unusable by either reader were subsequently removed from the study. This resulted in a reduction to 99 samples for the left side and 97 for the right. The reasons for these exclusions were the presence of stents, bypasses, or significant artefacts caused by implants.

Different class scenarios were generated for various class separations, including binary class (0 *versus* 1, 2 or 3), three class (0 *versus* 1 or 2 *versus* 3), and four-class (0-to-3) cases, to differentiate between the different steno-occlusion severity levels. The right and left sides were examined separately.

If a patient received a DSA examination within 30 days after the MRA, as represented in Fig. 1, additional labels were recorded using the radiological reports and a consensus reading by the radiologists who labelled the MRA data. Due to the limited data samples for the labels derived from DSA, we applied only a binary class separation according to the previously mentioned scheme, resulting in 29 samples for the left side and 35 for the right side. Since DSA was the reference standard, these labels were treated as the ground truth.

### Neural network

In this study, an EfficientNet B0 [12] implementation was used to perform arterial steno-occlusion detection using deep learning techniques. The implementation was carried out using the PyTorch Lightning framework [13], which provides a high-level interface for building and training deep learning models.

EfficientNet is a convolutional neural network (CNN) designed for high accuracy with fewer parameters and computational resources. It uses a compound scaling method to balance the trade-off between depth, width, and resolution. EfficientNet outperforms previous models on benchmark datasets while using less resources [12].

To train the model, most of the pre-trained models from Nguyen et al. [11] were used for the three and four-class problems. However, since the current study is focused on the binary data split (steno-occlusion *versus* non-steno-occlusion), a new model had to be trained to fit the new class separation using the training regime and separate dataset from Nguyen et al. [11]. For each data sample, 13 MIP images reconstructed along different angles were fed as channels to the CNN.

### Analysis

To evaluate the inter-reader agreement on the steno-occlusion status of the arteries, a quadratic weighted Cohen κ was utilised. This metric measures the level of agreement between the two readers and the model, considering both the extent and direction of disagreement between the readers [14].

For cases where the ground truth was obtained through DSA, the model’s performance was tested. Accuracy, which measures the proportion of correct predictions in the total predictions made, and the area under the curve at receiver operating characteristics analysis (ROC-AUC), representing the model’s ability to distinguish between positive and negative classes, were both calculated [15].

Beyond the quantitative evaluation metrics, occlusion mapping was performed on a subset of 5 samples to visually assess the deep learning model’s performance. Occlusion mapping in DL involves selectively blocking or masking parts of an input image and observing the resulting changes in the network’s predictions. This process helps understand which regions or features are most influential in the network’s decision-making process [16].

The limitation to a small number of samples was due to the need for fine-tuning the occlusion mapping parameters individually for different combinations of trained CNNs and data samples.

## Results

**Fig. 2:**
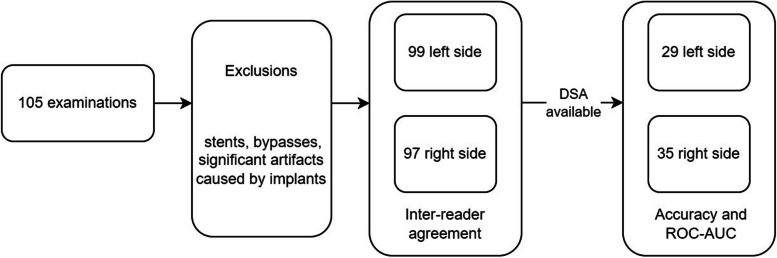
Flowchart of the number of data samples for the different steps. *ROC-AUC* Area under the curve at receiver operating characteristics analysis

The different number of data samples for each experiment after certain samples were excluded are presented in Fig. 2. Distinct representations are provided for both the left and right sides.

The results revealed a high level of inter-reader agreement between the two radiologists, as evidenced by the quadratic weighted Cohen κ scores of ≥ 0.90 and ≥ 0.87 for the left and right side, respectively (Table 2), across all class separations. Conversely, the agreement between the radiologists and the automatic DL model was lower, with scores of ≥ 0.72 and ≥ 0.66 for the left and right side, respectively (Table 2), across all class separations [17].

**Table 2:** Tabular results of the quadratic weighted Cohen κ scores for the different readers and the deep learning (DL) model

Side	Classes	Reader 1 *versus* Reader 2	Reader 1 *versus* DL model	Reader 2 *versus* DL model
Left	2	0.90	0.72	0.74
	3	0.91	0.78	0.73
	4	0.92	0.80	0.73
Right	2	0.87	0.70	0.69
	3	0.87	0.69	0.69
	4	0.89	0.66	0.68

Different class scenarios were generated for various class separations, including binary class (0 *versus* 1−3), three-class (0 *versus* 1 or 2 *versus* 3), and four-class (0−3) cases

**Fig. 3:**
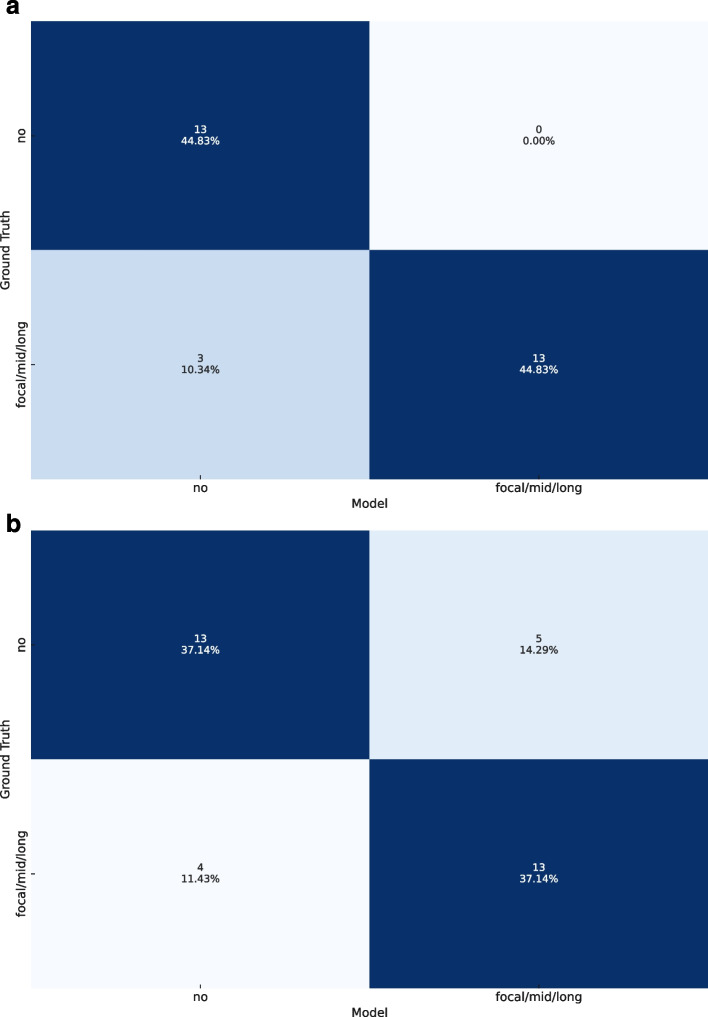
Confusion matrices representing the predictions of the model using the labels derived from digital subtraction angiography as the ground truth. Results for the left side (**a**) and for the right side (**b**)

The model’s predictions for the data samples, which utilised consensus reading labels derived from DSA as ground truth, demonstrated high accuracy as depicted in Fig. 3. Misclassifications were minimal, evidenced by an accuracy of 0.897 and ROC-AUC of 0.913 for the left side. These metrics align closely with the labelling done by radiologists based on radial MIP reconstructions. Overall, the performance on the left side was slightly superior to that on the right side, as detailed in Table 3.

**Table 3:** Results of the predictions of the deep learning (DL) model and readers using the labels derived from digital subtraction angiography as the ground truth

Reader	Side	Accuracy	ROC-AUC
DL model	Left	0.897	0.913
	Right	0.743	0.830
Reader 1	Left	0.931	0.930
	Right	0.800	0.799
Reader 2	Left	0.862	0.861
	Right	0.771	0.771

*ROC-AUC* Area under the curve at receiver operating characteristics analysis

**Fig. 4:**
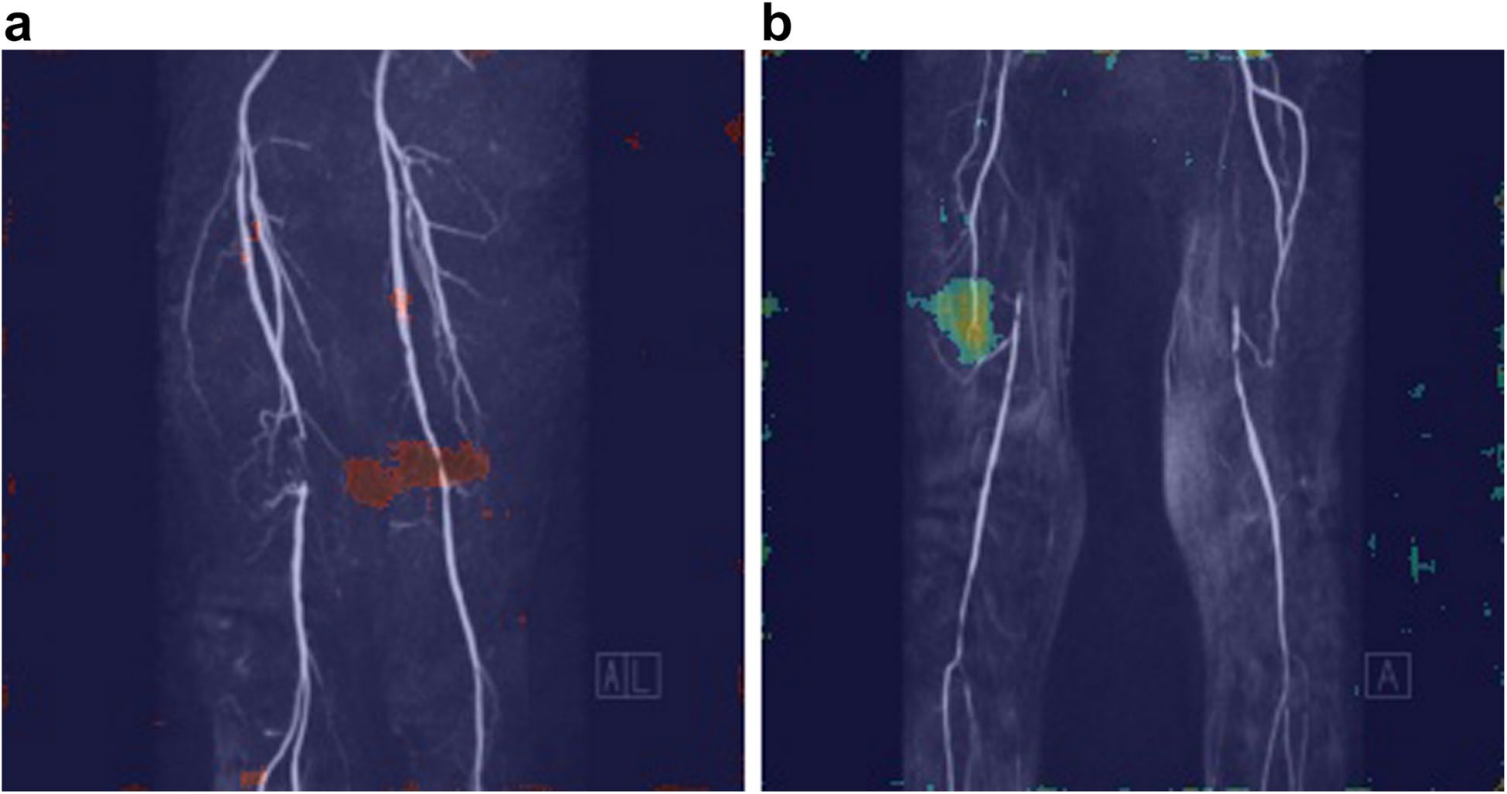
Exemplary occlusion map, heatmap overlaid on the input image, depicts a neural network trained to detect arterial steno-occlusions on the left side (**a**) and the right side (**b**). The overlaid colour intensity indicates which areas of the image were most important to the neural network for its classification task

Regarding the exemplary occlusion maps created for 5 samples, visually evaluated by the radiologists who labelled the data to assess the accuracy of the model’s predictions, as exemplary shown in Fig. 4, this qualitative evaluation was found to be consistent with the quantitative metrics, playing in favour that the model was able to detect the correct side and the approximate area of the occlusions.

## Discussion

This study suggests that the AI, using the tested DL model, was effective in detecting arterial steno-occlusions of the superficial femoral and popliteal artery on MRA in PAD patients within a clinical dataset. The inter-reader agreement between radiologists and the DL model was high. However, the agreement did not surpass that observed between two radiologists. Despite this relatively lower level of agreement, it is still considered to be a good degree of concordance [17]. This indicates that while the DL model is effective, it has not yet surpassed human expertise in this domain.

Additionally, an effort was made to refine class separation to differentiate based on the length of stenosis, providing valuable information for clinical treatment decisions [18]. However, this more refined class separation was not applied in the experiments involving the DSA subset due to the limited number of data samples. Consequently, an in-depth discussion on the impact of the additional information from the class separation is not feasible at this stage. Therefore, further optimisation of the method is recommended before considering its routine clinical application.

When applied to the subdataset using DSA as the ground truth, the model’s performance was comparable to that of radiologists, suggesting it can detect real steno-occlusions on par with a radiologist. The results for the left side in the binary case in this study are consistent with the findings reported by Nguyen et al. [11], with an accuracy of 0.897 and a ROC-AUC of 0.913, compared to 0.851 and 0.917 as reported by Nguyen et al. [11]. Notably, the results of the subdataset are based on a consensus reading using DSA, which is considered the references standard. Additionally, their method employed only one reader for data labelling, which could introduce potential bias.

Dai et al. [10] reported slightly better results with an accuracy of 0.915 and a ROC-AUC of 0.987 [10]. However, their methodology depended on segmented areas of axial CT slices, which may be less practical in a clinical setting since this method requires additional pre-processing steps. Moreover, this technique does not capture information about the length of the steno-occlusions when using the CNN, necessitating further pre-processing steps for segmentation. Of note, the length of the steno-occlusions is a critical factor in determining different treatment approaches.

Calcification, a frequent element in steno-occlusions, can create difficulties in CT imaging because of its high attenuation values. Such calcified plaques can produce blooming artefacts in CT scans, potentially leading to an overestimation of steno-occlusion severity. In contrast, MRA is unaffected by these artefacts and can provide a clearer depiction of the vessel lumen.

A challenge that DL models encounter in the medical field is the “black box” design, where the user cannot evaluate whether the model is accurately measuring the intended target, such as arterial steno-occlusions in our study, or if it is relying on some other image feature introduced by bias in the training and testing data [19]. To address this issue, we conducted occlusion mapping on a small subsample of data and analysed it qualitatively. The results suggest that the neural network correctly identifies the area of arterial steno-occlusion occurrence.

This study has several limitations. We focused solely on classifying relevant steno-occlusions based on their length and distribution. In clinical settings, however, a more nuanced classification is practised, which takes into account different degrees of steno-occlusions in percentage terms.

Manual labelling by radiologists, especially for extensive datasets, can be both time-intensive and resource-heavy. Such a process often results in challenges with class separation and might lead to the omission of certain findings. Transitioning to structured reporting over prose text could alleviate this, as structured reports are more amenable to automatic label extraction for DL models [20]. Additionally, labels are often readily accessible through free-form radiological reports. The adoption of advanced transformer-based models, like ImageBERT [21], which can handle both textual and visual data, could streamline this process and enable training on more expansive datasets.

Currently, our model is specifically designed for detecting steno-occlusions in the superficial femoral and popliteal arteries, as visualised in radial MIP images. It does not yet have the capability to identify other vascular structures or pathologies, such as bypass grafts, nor does it analyse the pelvic arteries and lower extremities, which present more complex challenges and likely require additional training data. Additionally, the model has not been tested with other imaging modalities, such as CTA. These limitations present opportunities for further research and development.

In conclusion, our findings suggest that, with further refinement, the proposed DL model holds promise as an effective tool for assisting in the detection of arterial steno-occlusions in patients with PAD. Although the model demonstrates robust performance in the subset using DSA as the benchmark, it has not yet exceeded the expertise of human radiologists. This is underscored by the increased inter-reader agreement observed among radiologists. Moreover, the current applicability of the model is restricted to the upper legs and does not include certain artefacts. This task is relatively straightforward for radiologists, who do not require an assistive tool, as demonstrated by the high inter-reader agreement. However, a more advanced version of the tool could potentially reduce the workload for radiologists and improve patient outcomes by offering enhanced decision support.

We recommended that further technical enhancements be pursued to meet daily clinical needs. This includes classifying various degrees of steno-occlusions by percentage and expanding coverage to abdominal, pelvic, and below-the-knee arterial regions.

## Data Availability

The datasets analysed during the current study are not publicly available due to data privacy of patient data but are available from the corresponding author on reasonable request.

## Abbreviations

AI: Artificial intelligence
CNN: Convolutional neural network
CT: Computed tomography
CTA: Computed tomography angiography
DL: Deep learning
DSA: Digital subtraction angiography
MIP: Maximum intensity projection
MRA: Magnetic resonance angiography
PAD: Peripheral arterial disease
ROC-AUC: Area under the curve at receiver operating characteristics analysis
